# Assessing the impact of intervention strategies against *Taenia solium* cysticercosis using the EPICYST transmission model

**DOI:** 10.1186/s13071-017-1988-9

**Published:** 2017-02-09

**Authors:** Peter Winskill, Wendy E. Harrison, Michael D. French, Matthew A. Dixon, Bernadette Abela-Ridder, María-Gloria Basáñez

**Affiliations:** 1 Medical Research Council Centre for Outbreak Analysis and Modelling, Department of Infectious Disease Epidemiology, School of Public Health, Faculty of Medicine, Imperial College London, St Mary’s Campus, London, W2 1PG UK; 2 Schistosomiasis Control Initiative, Department of Infectious Disease Epidemiology, School of Public Health, Faculty of Medicine, Imperial College London, St. Mary’s Campus, London, W2 1PG UK; 3 Department of Infectious Disease Epidemiology, School of Public Health, Faculty of Medicine, Imperial College London, St Mary’s Campus, London, W2 1PG UK; 4 Neglected Zoonotic Diseases Team, Department of Neglected Tropical Diseases (NTD), World Health Organization (WHO), 20 Avenue Appia, 1211, Geneva 27, Switzerland; 5 London Centre for Neglected Tropical Disease Research, Department of Infectious Disease Epidemiology, School of Public Health, Faculty of Medicine, Imperial College London, St Mary’s Campus, London, W2 1PG UK; 6 Current address: Research Triangle Institute, 701 13st Street NW, Washington D.C., 20005 USA

**Keywords:** Cysticercosis, Neurocysticercosis, Taeniasis, *Taenia solium*, Tapeworm, Intervention, Mathematical modelling, EPICYST, Latin-hypercube sampling, Partial rank correlation coefficient index, Basic reproduction number

## Abstract

**Background:**

The pork tapeworm, *Taenia solium*, and associated human infections, taeniasis, cysticercosis and neurocysticercosis, are serious public health problems, especially in developing countries. The World Health Organization (WHO) has set goals for having a validated strategy for control and elimination of *T. solium* taeniasis/cysticercosis by 2015 and interventions scaled-up in selected countries by 2020. Timely achievement of these internationally-endorsed targets requires that the relative benefits and effectiveness of potential interventions be explored rigorously within a quantitative framework.

**Methods:**

A deterministic, compartmental transmission model (EPICYST) was developed to capture the dynamics of the taeniasis/cysticercosis disease system in the human and pig hosts. Cysticercosis prevalence in humans, an outcome of high epidemiological and clinical importance, was explicitly modelled. A next generation matrix approach was used to derive an expression for the basic reproduction number, *R*_0_. A full sensitivity analysis was performed using a methodology based on Latin-hypercube sampling partial rank correlation coefficient index.

**Results:**

EPICYST outputs indicate that chemotherapeutic intervention targeted at humans or pigs would be highly effective at reducing taeniasis and cysticercosis prevalence when applied singly, with annual chemotherapy of humans and pigs resulting, respectively, in 94 and 74% of human cysticercosis cases averted. Improved sanitation, meat inspection and animal husbandry are less effective but are still able to reduce prevalence singly or in combination. The value of *R*_0_ for taeniasis was estimated at 1.4 (95% Credible Interval: 0.5–3.6).

**Conclusions:**

Human- and pig-targeted drug-focussed interventions appear to be the most efficacious approach from the options currently available. The model presented is a forward step towards developing an informed control and elimination strategy for cysticercosis. Together with its validation against field data, EPICYST will be a valuable tool to help reach the WHO goals and to conduct economic evaluations of interventions in varying epidemiological settings.

**Electronic supplementary material:**

The online version of this article (doi:10.1186/s13071-017-1988-9) contains supplementary material, which is available to authorized users.

## Background

The persistence of infections by the pork tapeworm, *Taenia solium*, and the associated conditions of cysticercosis and neurocysticercosis remain a major public health concern, especially in impoverished communities that are highly dependent on pig farming [1]. The roadmap set out by the World Health Organization (WHO) in 2012 to overcome the impact of 17 prioritised neglected tropical diseases (NTDs) called for a validated strategy, by 2015, for the control and elimination of *T. solium* taeniasis and cysticercosis. This roadmap also called for the implementation of scaled-up interventions with the aim of control and elimination in selected countries by 2020 [2]. Neurocysticercosis remains one of the foremost causes of epilepsy in developing countries [3]; a recent systematic review estimated that 29.0% [95% confidence intervals (95% CI): 22.9–35.5%] of people with epilepsy were also afflicted with neurocysticercosis [4].

The multi-host life-cycle of *T. solium* and complications associated with humans becoming accidental dead-end hosts - resulting in the development of cysticercosis and, in some cases, the potentially fatal neurocysticercosis - lead to complex transmission dynamics. The various stages of the life-cycle, through the pig and human hosts, as well as the interface between parasite eggs and the environment provide a wide range of possible targets for control interventions. Whilst a variety of potential interventions have shown promise in their ability to control taeniasis and cysticercosis, there remains a number of challenges that must be overcome to facilitate the control and elimination of taeniasis and cysticercosis as public health problems and help the WHO to achieve its 2020 goals [5]. These include further development of new drugs and accessible diagnostics in humans, conducting randomised clinical field trials to assess pig-focussed strategies such as drug treatment and vaccination, implementing a progression of behaviour-change interventions, and building transmission models to assess intervention strategies [6].

Current intervention strategies targeted at the pig-stage of the life-cycle include chemotherapeutic treatment and vaccination. The development and field-testing of vaccines that protect pigs from porcine cysticercosis are promising. A number of potential vaccines has been developed, with the S3Pvac [7, 8] and TSOL18 [9, 10] vaccines being the most encouraging, field evaluated, candidates. A small field trial of the TSOL18 vaccine in Cameroon demonstrated that the vaccine could be highly effective in the field [11]. A second option to target the infection in the intermediate host is anthelmintic chemotherapy of pigs. The most likely candidate for this intervention strategy is oxfendazole, which has performed well at controlling the parasite in pigs [12–14] and has also been shown to be effective in the field [15]. However, treatment using oxfendazole can lead to necrotic lesions causing issues when consuming or selling treated pork that harboured a heavy cyst burden [16].

Anthelmintic treatment of humans is an attractive option. Indeed, human treatment, with praziquantel or niclosamide, has been proposed and trialled with some success [17–21]. However, to date, there has not been wide scale roll out of easily accessible, affordable and reliable diagnostic tests to discriminate between taeniasis and cysticercosis infection. Diagnostics are important because treatment with praziquantel can be a potential cause of severe adverse effects (SAEs) as the drug is cysticidal and can cause inflammation around dying cysts in those with cysticercosis. Praziquantel therapy at high doses in people harbouring a large number of occult cysticerci may lead to severe neurological outcomes [22]. This raises some concerns for schistosomiasis control programmes that deliver praziquantel at large scale in areas where the co-endemicity with *T. solium* is unknown. The impact of praziquantel on a community co-endemic for schistosomiasis and *T. solium* is the subject of an ongoing study in Malawi. The concerns about praziquantel-induced SAEs, combined with high logistical costs have led the focus of human preventative chemotherapy (PCT) away from mass drug administration (MDA) [16], a strategy otherwise endorsed by the WHO for the control of human helminthiases.

A number of behavioural, educational and infrastructural interventions target a variety of points throughout the life-cycle of the parasite. Meat inspection, to screen out infected pork, is one option, although a lack of sensitivity in visual examination, poorly implemented practice and avoidance of such schemes by farmers and pig traders have been problematic in past efforts [23]. Changes to pig-husbandry practices such as indoor-rearing or corralling, aimed at limiting the contact between pigs and the infectious agent (eggs) in the environment, have been proposed but have proven hard to implement in resource-limited settings [24]. A field trial of a health-education intervention in Tanzania has demonstrated how difficult it is to instigate large impacts on taeniasis or cysticercosis infection levels through behavioural change [25]. Public education interventions have been shown to be efficacious although their sustainability has not been ascertained [25]. Improved sanitation infrastructure may be effective but is reliant, in part, on economic factors out of the control of a specific intervention effort.

Mathematical modelling of the transmission dynamics of *T. solium* has the potential to be a powerful tool with which the problem of control and elimination of taeniasis and cysticercosis can be interrogated. As for many NTDs, and specifically helminth infections, there is much scope for the further development, improved parameterisation, implementation and validation of transmission models that allow intervention impacts to be accurately assessed [26, 27]. As well as providing a tool for evaluating interventions, mathematical models may also be used to identify key knowledge gaps and uncertainties which must be addressed to further our understanding of the system.

Two previously published models of *T. solium* transmission dynamics have approached the problem from both the deterministic [28] and stochastic [29] standpoints. The Reed-Frost framework presented by Kyvsgaard et al. [28] is the single published reference point for deterministic modelling of this system. This model was used to explore *T. solium* transmission dynamics and to assess a range of interventions. We have built upon the foundation laid down by this model and present EPICYST, a (deterministic) full transmission model capturing the life-cycle of *T. solium* as well as the dynamics of taeniasis and cysticercosis in the human population, of cysticercosis in the porcine population, and of *T. solium* eggs in the environment. The EPICYST model is also used to derive an expression for the basic reproduction number, *R*_0_, of the infection. Derivations of *R*_0_ for helminths have focussed on dioecious (separate sexes) species using infection intensity frameworks [30], but *T. solium* is hermaphrodite and a prevalence framework here is appropriate. We illustrate our results by assessing the projected impact of a suite of interventions and combinations of interventions against cysticercosis. Uncertainties and knowledge gaps are rigorously explored with a full sensitivity analysis.

## Methods

### The EPICYST model

**Fig. 1:**
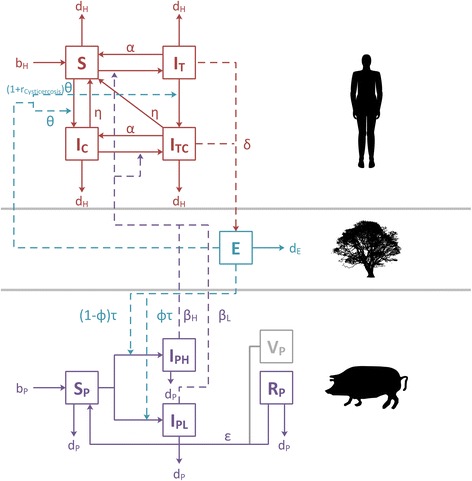
Flow diagram of the transmission model. State variables are represented by compartments; flows or rates associated with model parameters by arrows. The three model sections from top to bottom represent, respectively, human compartments, the environmental compartment and pig compartments. (The silhouettes of the human, pig and tree are from https://commons.wikimedia.org/)

Our deterministic, compartmental model tracks the host populations (humans, pigs) infected with the parasite as well as the parasite transmission stages (gravid proglottides/eggs) in the environment. Specific compartments are included to model the prevalence of cysticercosis in humans. A diagram of the model, associated parameters and parameter values are shown in Fig. 1, Tables 1 and 2, respectively.

**Table 1:** Definition of variables and parameters used in the transmission model

State variables	Description	
*S*	Susceptible humans	
*I*_*T*_	Humans with taeniasis only	
*I*_*C*_	Humans with cysticercosis only	
*I*_*TC*_	Humans with taeniasis and cysticercosis	
*S*_*P*_	Susceptible pigs	
*I*_*PL*_	Pigs with cysticercosis (low cyst burden)	
*I*_*PH*_	Pigs with cysticercosis (high cyst burden)	
*R*_*p*_	Recovered (immune) pigs	
*V*_*p*_	Vaccinated pigs	
*E*	*Taenia solium* eggs (in the environment)	
Demographic rates	Description	Units
*b*_*H*_	Human births (net rate, set to maintain stable population size)	Month^-1^
*d*_*H*_	Human death rate (per capita)	Month^-1^
*b*_*P*_	Pig births (net rate, set to maintain stable population size)	Month^-1^
*d*_*P*_	Pig death rate (per capita)	Month^-1^
*d*_*E*_	Egg removal/death rate (per capita)	Month^-1^
Transmission parameters	Description	Units
*α*	Human recovery rate from taeniasis (reciprocal of the average lifespan of the adult *Taenia solium* worm)	Month^-1^
*τ*	Egg to pig transmission coefficient (product of the contact rate and the probability of infection upon consumption of a *Taenia solium* egg)	Month^-1^
*ϕ*	Proportion of pigs with low cyst burden; (1-*ϕ*) is the proportion of pigs with high cyst burden	–
*δ*	Egg production rate	Month^-1^
*ε*	Rate of loss of immunity in pigs	Month^-1^
*χ*	Rate of human pork meal procurement	Month^-1^
*π*_*L*_	Probability of human becoming infected having consumed infected pork with a low cyst burden	–
*r*_consumption_	Risk multiplier of discarding high cyst-burden pork (relative to discarding low cyst-burden pork)	–
*r*_infection_	Risk multiplier of infection on consuming high cyst-burden pork (relative to consuming low cyst-burden pork)	–
*π*_*H*_	Probability of human becoming infected having consumed infected pork with a high cyst burden, *π*_*H*_ *= π*_*L*_(1 + *r*_consumption_ + *r*_infection_)	–
*β*_*L*_	Pig (low cyst burden) to human transmission coefficient (a product of the contact rate and the probability of infection upon consumption of infected pork, *β*_*L*_ = *χπ*_*L*_	Month^-1^
*β*_*H*_	Pig (high cyst burden) to human transmission coefficient (a product of the contact rate and probability of infection upon consumption of infected pork, *β*_*H*_ = *χπ*_*H*_	Month^-1^
*θ*	Egg to human cysticercosis transmission coefficient (a product of the contact rate and probability of infection upon consumption of *Taenia solium* eggs)	Month^-1^
*r*_Cysticercosis_	Risk multiplier for cysticercosis (applied to theta if the individual has taeniasis)	–
*η*	Human recovery rate from cysticercosis (reciprocal of the average duration of cysticercosis infection in humans)	Month^-1^

**Table 2:** Parameter values for cysticercosis model (values are expressed per month). Minimum and maximum values were used for the sensitivity analysis

Parameter	Value	Minimum	Maximum	Derivation (min, max)	Reference
Human population size	10,000	–	–	–	–
Pig population size	2000	–	–	–	–
*d*_*H*_	0.0015	0.0013	0.0018	Average human life expectancy of 54 years (46, 64)	[56]
*d*_*P*_	0.083	0.042	0.17	Average age at slaughter: 1 year (0.5, 2)	[14, 28, 57]
*d*_*E*_^a^	2	0.083	4	Average period of persistence of eggs in environment: 2 weeks (1 week, 3 months)	[50–53, 58]
*α*	0.042	0.017	0.17	Average lifespan of *T. solium*: 2 years (0.5, 5)	[59, 60]
*τ* ^*a*^	2.36 × 10^-10^	0.25*τ*	4*τ*	–	^b^
*ϕ*	0.8	0.1	0.9	–	[61]
*δ*	960,000	640,000	1,800,000	–	[60, 62]
*ε*	0.01	0.0075	0.0125	–	[63]
*χ*	0.5	0.083	0.68	Assumes 1 pork meal = 200 g; 1.2 kg per capita per annum (0.2 kg, 3.4 kg) = an average 6 pork meals per year	[64]
*π*_*L*_^a^	0.0084	0.25*π*_*L*_	4*π*_*L*_	–	^b^
*r*_consumption_	-0.25	-0.66	0	–	^c^
*r*_infection_	1	0	2	–	^c^
*θ* ^a^	2.45 × 10^-11^	0.25*θ*	4*θ*	–	^b^
*r*_Cysticercosis_	1	0	4	–	[65]
*η*	0.0277	0.00167	0.083	Average duration of cysticercosis infection in humans: 3 years (1, 50)	[66]

^a^Indicates that a uniform distribution was specified for the LHS-PRCC, a triangle distribution was used for all other parameters

^b^Parameter values were derived by equilibrium analysis

^c^Where evidence in the literature was scarce to motivate parameter values, the values chosen were based upon expert opinion of plausible and conservatively wide ranges and their influence was further explored in the sensitivity analysis

#### Assumptions

EPICYST has a number of simplifying assumptions. Both human and pig population sizes remain constant throughout. We assume that humans do not develop immunity to reinfection. Humans become infected/infectious with taeniasis (i.e. the pre-patent period of 5–10 weeks before shedding eggs is very short in comparison with the 55 years assumed human lifespan and can, therefore, be mathematically omitted). There is no excess mortality in humans with cysticercosis. We also assume that pigs do not naturally clear the infection (there is no natural recovery of pigs or the recovery rate of pigs is very slow in comparison with their death rate and can be omitted). The recovered/immune compartment is, therefore, included for pigs assumed to become immune after treatment. We also assume that the number of eggs consumed by pigs and humans has a negligible effect on the total number of eggs present in the environment. The distribution of cysticerci in pigs is assumed to be aggregated or overdispersed [31]. Although we do not model explicitly this distribution, the heterogeneity of cyst burden in the pig population is implemented by including two infected compartments for pigs harbouring low and high cyst burdens (${\mathit{\mathsf{I}}}_{\mathit{\mathsf{PL}}}$ and ${\mathit{\mathsf{I}}}_{\mathit{\mathsf{PH}}}$ respectively).

#### Model equations

The rates of change with respect to time in the number of susceptible humans (*S*); humans infected only with taeniasis $\left({\mathit{\mathsf{I}}}_{\mathit{\mathsf{T}}}\right)$: harbouring the adult stage of *T. solium* and therefore acting as definitive hosts; humans infected only with cysticercosis $\left({\mathit{\mathsf{I}}}_{\mathit{\mathsf{c}}}\right)$: harbouring the larval stage and therefore becoming dead-end ‘intermediate’ hosts; and humans with both taeniasis and cysticercosis $\left({\mathit{\mathsf{I}}}_{\mathit{\mathsf{TC}}}\right)$ in the human population are given by the following ordinary differential equations (ODEs),

1 $$ \frac{\mathit{\mathsf{d}\mathsf{S}}}{\mathit{\mathsf{d}\mathsf{t}}}={\mathit{\mathsf{b}}}_{\mathit{\mathsf{H}}}+\alpha {\mathit{\mathsf{I}}}_{\mathit{\mathsf{T}}}+\eta {\mathit{\mathsf{I}}}_{\mathit{\mathsf{C}}}+\eta {\mathit{\mathsf{I}}}_{\mathit{\mathsf{T}\mathsf{C}}}-\frac{\beta_{\mathit{\mathsf{L}}}\mathit{\mathsf{S}}{\mathit{\mathsf{I}}}_{\mathit{\mathsf{P}\mathsf{L}}}}{{\mathit{\mathsf{N}}}_{\mathit{\mathsf{P}}}}-\frac{\beta_{\mathit{\mathsf{H}}}\mathit{\mathsf{S}}{\mathit{\mathsf{I}}}_{\mathit{\mathsf{P}\mathsf{H}}}}{{\mathit{\mathsf{N}}}_{\mathit{\mathsf{P}}}}-\theta \mathit{\mathsf{S}}\mathit{\mathsf{E}}-{\mathit{\mathsf{d}}}_{\mathit{\mathsf{H}}}\mathit{\mathsf{S}} $$

2 $$ \frac{\mathit{\mathsf{d}}{\mathit{\mathsf{I}}}_{\mathit{\mathsf{T}}}}{\mathit{\mathsf{d}\mathsf{t}}}=\frac{\beta_{\mathit{\mathsf{L}}}\mathit{\mathsf{S}}{\mathit{\mathsf{I}}}_{\mathit{\mathsf{P}\mathsf{L}}}}{{\mathit{\mathsf{N}}}_{\mathit{\mathsf{P}}}}+\frac{\beta_{\mathit{\mathsf{H}}}\mathit{\mathsf{S}}{\mathit{\mathsf{I}}}_{\mathit{\mathsf{P}\mathsf{H}}}}{{\mathit{\mathsf{N}}}_{\mathit{\mathsf{P}}}}-\left(\alpha +\left(\mathsf{1}+\mathit{\mathsf{r}}\right)\theta \mathit{\mathsf{E}}+{\mathit{\mathsf{d}}}_{\mathit{\mathsf{H}}}\right){\mathit{\mathsf{I}}}_{\mathit{\mathsf{T}}} $$

3 $$ \frac{\mathit{\mathsf{d}}{\mathit{\mathsf{I}}}_{\mathit{\mathsf{C}}}}{\mathit{\mathsf{d}\mathsf{t}}}=\theta \mathit{\mathsf{S}}\mathit{\mathsf{E}}+\alpha {\mathit{\mathsf{I}}}_{\mathit{\mathsf{TC}}}-\frac{\beta_{\mathit{\mathsf{L}}}{\mathit{\mathsf{I}}}_{\mathit{\mathsf{C}}}{\mathit{\mathsf{I}}}_{\mathit{\mathsf{P}\mathsf{L}}}}{{\mathit{\mathsf{N}}}_{\mathit{\mathsf{P}}}}-\frac{\beta_{\mathit{\mathsf{H}}}{\mathit{\mathsf{I}}}_{\mathit{\mathsf{C}}}{\mathit{\mathsf{I}}}_{\mathit{\mathsf{P}\mathsf{H}}}}{{\mathit{\mathsf{N}}}_{\mathit{\mathsf{P}}}}-\left(\eta +{\mathit{\mathsf{d}}}_{\mathit{\mathsf{H}}}\right){\mathit{\mathsf{I}}}_{\mathit{\mathsf{C}}} $$

4 $$ \frac{\mathit{\mathsf{d}}{\mathit{\mathsf{I}}}_{\mathit{\mathsf{T}\mathsf{C}}}}{\mathit{\mathsf{d}\mathsf{t}}}=\frac{\beta_{\mathit{\mathsf{L}}}{\mathit{\mathsf{I}}}_{\mathit{\mathsf{C}}}{\mathit{\mathsf{I}}}_{\mathit{\mathsf{P}\mathsf{L}}}}{{\mathit{\mathsf{N}}}_{\mathit{\mathsf{P}}}}+\frac{\beta_{\mathit{\mathsf{H}}}{\mathit{\mathsf{I}}}_{\mathit{\mathsf{C}}}{\mathit{\mathsf{I}}}_{\mathit{\mathsf{P}\mathsf{H}}}}{{\mathit{\mathsf{N}}}_{\mathit{\mathsf{P}}}}+\left(\mathsf{1}+\mathit{\mathsf{r}}\right)\theta {\mathit{\mathsf{I}}}_{\mathit{\mathsf{T}}}\mathit{\mathsf{E}}-\left(\alpha +\eta +{\mathit{\mathsf{d}}}_{\mathit{\mathsf{H}}}\right){\mathit{\mathsf{I}}}_{\mathit{\mathsf{T}\mathsf{C}}}, $$

where *b*_*H*_ is the net number of humans births; and *η* are the per capita human recovery rates from taeniasis and cysticercosis, respectively; *β*_*L*_ and *β*_*H*_ are the pig to human transmission coefficients for pigs with low and high cyst burdens, respectively; *N*_*P*_ represents the pig population size; *θ* is the egg to human cysticercosis transmission coefficient (the transmission coefficients being the product of the rate of contact and the transmission probability upon contact); *r* (listed as *r*_Cysticercosis_ in Figure 1 and Table 1) is a risk multiplier for cysticercosis in those with taeniasis (to adjust for increased risk due to autoinfection), and *d*_*H*_ the per capita human mortality rate.

Human contact rate with pork is assumed to be frequency dependent whilst human and pig contact rates with eggs in the environment are assumed to be density dependent [32]. The rate of change in the number of infectious eggs present in the environment (*E*) with respect to time is

5 $$ \frac{\mathit{\mathsf{d}\mathsf{E}}}{\mathit{\mathsf{d}\mathsf{t}}}=\delta \left({\mathit{\mathsf{I}}}_{\mathit{\mathsf{T}}}+{\mathit{\mathsf{I}}}_{TC}\right)-{\mathit{\mathsf{d}}}_{\mathit{\mathsf{E}}}\mathit{\mathsf{E}}, $$

where *δ* is the egg production rate into the environment, and *d*_*E*_ the egg loss rate from the environment (capturing physical removal or loss of infectiousness/viability).

The rates of change with respect to time in the number of susceptible pigs (*S*_*P*_), the number of infected pigs harbouring a low cyst burden (*I*_*PL*_), the number of infected pigs harbouring a high cyst burden (*I*_*PH*_) and the number of immune (post-treatment (*R*_*P*_) or due to vaccination (*V*_*P*_)) pigs in the pig population (the intermediate hosts) are given in the following ODEs,

6 $$ \frac{\mathit{\mathsf{d}}{\mathit{\mathsf{S}}}_{\mathit{\mathsf{P}}}}{\mathit{\mathsf{d}\mathsf{t}}}={\mathit{\mathsf{b}}}_{\mathit{\mathsf{P}}}+\varepsilon {\mathit{\mathsf{R}}}_{\mathit{\mathsf{P}}}-\phi \tau {\mathit{\mathsf{S}}}_{\mathit{\mathsf{P}}}\mathit{\mathsf{E}}-\left(\mathit{\mathsf{1}}-\phi \right)\tau {\mathit{\mathsf{S}}}_{\mathit{\mathsf{P}}}\mathit{\mathsf{E}}-{\mathit{\mathsf{d}}}_{\mathit{\mathsf{P}}}{\mathit{\mathsf{S}}}_{\mathit{\mathsf{P}}} $$

7 $$ \frac{\mathit{\mathsf{d}}{\mathit{\mathsf{I}}}_{\mathit{\mathsf{P}\mathsf{L}}}}{\mathit{\mathsf{d}\mathsf{t}}}=\phi \tau {\mathit{\mathsf{S}}}_{\mathit{\mathsf{P}}}\mathit{\mathsf{E}}-{\mathit{\mathsf{d}}}_{\mathit{\mathsf{P}}}{\mathit{\mathsf{I}}}_{\mathit{\mathsf{P}\mathsf{L}}} $$

8 $$ \frac{\mathit{\mathsf{d}}{\mathit{\mathsf{I}}}_{\mathit{\mathsf{P}\mathsf{H}}}}{\mathit{\mathsf{d}\mathsf{t}}}=\left(1-\phi \right)\tau {\mathit{\mathsf{S}}}_{\mathit{\mathsf{P}}}\mathit{\mathsf{E}}-{\mathit{\mathsf{d}}}_{\mathit{\mathsf{P}}}{\mathit{\mathsf{I}}}_{\mathit{\mathsf{P}\mathsf{H}}} $$

9 $$ \frac{\mathit{\mathsf{d}}{\mathit{\mathsf{R}}}_{\mathit{\mathsf{P}}}}{\mathit{\mathsf{d}\mathsf{t}}}=-\left(\varepsilon +{\mathit{\mathsf{d}}}_{\mathit{\mathsf{P}}}\right){\mathit{\mathsf{R}}}_{\mathit{\mathsf{P}}} $$

10 $$ \frac{\mathit{\mathsf{d}}{\mathit{\mathsf{V}}}_{\mathit{\mathsf{P}}}}{\mathit{\mathsf{d}\mathsf{t}}}=-\left(\varepsilon +{\mathit{\mathsf{d}}}_{\mathit{\mathsf{P}}}\right){\mathit{\mathsf{V}}}_{\mathit{\mathsf{P}}}, $$

where *b*_*P*_ is the net number of pig births; *ε* is the rate of loss of immunity; *ϕ* is the proportion of infected pigs that harbour low cyst burdens; *τ* is the egg to pig transmission coefficient and *d*_*P*_ the per capita pig mortality rate.

The pig to human transmission coefficients can be expressed as the product of the rate of contact and infection probability,

11 $$ {\beta}_L\phi +{\beta}_H\left(1-\phi \right)=\left(\chi {\pi}_L\right)\phi +\chi {\pi}_L\left(1+{\mathit{\mathsf{r}}}_{\mathsf{comsumption}}+{\mathit{\mathsf{r}}}_{\mathsf{infection}}\right)\left(1-\phi \right), $$

where, *χ* represents the rate of obtaining a pork meal; *π*_*L*_ is the probability of becoming infected once infected pork with low cyst burden is consumed; and r_consumption_ and *r*_infection_ are the risk multipliers quantifying, respectively, the decreased chance of consumption but increased chance of infection once pork is consumed with a high cyst burden, relative to pork with a low cyst burden (i.e., *π*_*H*_ = *π*_*L*_ *+ π*_*L*_ *r*_consumption_ + *π*_*L*_ *r*_infection_). The model was coded and run in R [33] using the *Odin* package [34].

A baseline scenario, with a human population of 10,000 and a pig population of 2000 was initiated to run at stable equilibrium (endemic state) to represent a sub-Saharan African setting (prevalence of taeniasis in humans = 2%, prevalence of cysticercosis in humans = 7% and prevalence of cysticercosis in pigs = 20% [35]). The model was then run at baseline for 30 years, after which single interventions or pairwise combinations of interventions were simulated for a further 20 years. The primary outcome measure of interest was the cumulative number of human cysticercosis cases averted over the period of the intervention.

### Interventions

EPICYST was used to assess the impact on infection prevalence in humans and pigs of various interventions applied singularly or in combination. The six single interventions include two pig-focussed interventions: vaccination and mass drug administration (MDA); three behavioural/infrastructural interventions: improved animal husbandry, improved sanitation and improved meat inspection, as well as testing and treatment of humans (test & treat) with taeniasis and not cysticercosis. The implementation of each intervention is further detailed below.

#### Pig MDA

Annual treatment of a proportion of all infected pigs with a drug of given efficacy. Although MDA should encompass the whole pig population irrespective of infection status, we are not, at this stage, modelling the possible impact of the suggested ‘prophylactic’ effect of treatment, described in [34], on susceptible (uninfected) pigs. Therefore, MDA is simulated in the model by treating already infected pigs. Every 12 months a proportion of infected pigs are instantaneously transferred from the *I*_*PL*_ and *I*_*PH*_ compartments to the *S*_*P*_ or to the *R*_*P*_ compartment. The proportion transferred to the latter (0.9) is the product of multiplying the proportion treated (the assumed therapeutic coverage = 90%) × the anthelmintic efficacy (the cure rate = 99% [12]), motivated by a study which found that oxfendazole-treated pigs did not become re-infected for at least three months after treatment and suggested that this protection may extend for longer periods and cover the remaining lifetime of the pigs [36].

#### Pig vaccination

Annual vaccination of a proportion of all susceptible pigs with a vaccine of given efficacy. Every 12 months a proportion of susceptible pigs are instantaneously transferred from the *S*_*P*_ compartment to the *V*_*P*_ compartment. The proportion would be the product of multiplying the proportion vaccinated (the assumed vaccination coverage = 90%) × the vaccine efficacy (99%) [11]. However, this proportion was adjusted downwards to account for the fact that piglets may become infected/infectious before a full vaccination course (this would include any necessary boosters required) can be completed (at the age of 3 months). The proportion transferred to the *R*_*P*_ compartment was modified according to the following expression,

12 $$ \mathrm{Adjusted}\ \mathrm{proportion}=0.9\left[0.99\hbox{-} \left(0.99\kern0.24em \tau\;E\times 3\right)\right]=0.84 $$

with *τ* and *E* as described earlier and values given in Table 2. (For the nominal value of *τ* = 2.36 × 10^-10^ the number of eggs in the environment, *E* is 96 × 10^6^.) The efficacy of the vaccine in partially vaccinated pigs was assumed to be zero.

#### Improved animal husbandry

Improved management of pig movement to decrease probability of contact with *Taenia solium* eggs in the environment. An example of this intervention would be the corralling of pigs in areas not likely to be contaminated with human faecal material. Post implementation *τ* is decreased by 20% as a nominal value; this was varied in the sensitivity analysis (see below).

#### Improved sanitation

Improved sanitation infrastructure to lead to a decrease in the number of *Taenia solium* eggs entering the environment. An example of this intervention would be the installation and use of pit latrines. Post-implementation *δ* is decreased by 20% as a nominal value; this was varied in the sensitivity analysis (see below).

#### Improved meat inspection

Improvements to meat inspection practice to reduce the rate of consumption of infected pork. We assume that meat with a high cyst burden is more detectable than meat with a low cyst burden. Post implementation *β*_*L*_ is decreased by 20% and *β*_*H*_ is decreased by 40% as nominal values; these were varied in the sensitivity analysis (see below).

#### Test & treat

Testing for cysticercosis and/or taeniasis and treating cases with taeniasis only. This is a hypothetical human-targeted intervention, implemented under the assumption that accurate, economically-viable, point-of-care, discriminative diagnostics tests are widely available in future. A proportion of humans with taeniasis (and not with cysticercosis) are treated annually. Every 12 months a proportion of humans with taeniasis (and not with cysticercosis) are transferred from the *I*_*T*_ compartment to the *S* compartment. The proportion is the product of multiplying the following factors: assumed therapeutic coverage (90%) × sensitivity of test to *T. solium* (0.97) × specificity of the test to cysticercosis infection (0.98) × assumed drug efficacy (99%) [20, 21]. These values were varied in the sensitivity analysis (see below).

### Sensitivity analysis

To assess the sensitivity of the model output to the suite of parameters and the uncertainty surrounding their nominal values, a sensitivity analysis based on Latin-hypercube sampling partial rank correlation coefficient index (LHS-PRCC) was performed. The LHS was used to produce samples of plausible parameter values. Following methodology set out in [37] and covered by [38], the range of values for each parameter (from minimum to maximum plausible values) was divided into *N* equal intervals based upon a given distribution (e.g. uniform, triangle, normal). Each parameter had one value drawn from each interval, resulting in *N* sets of parameter values and permitting an efficient exploration of parameter space. The partial rank correlation coefficient (PRCC) is a measure of the non-linear but monotonic relationship between a given parameter and model output, adjusted for the effects of all other parameters in the model. Given two paired variables, $\mathit{\mathsf{x}}$ and $\mathit{\mathsf{y}},$ and their respective sample means, $\overline{\mathit{\mathsf{x}}}$ and $\overline{\mathit{\mathsf{y}}}$, a standard correlation coefficient, *ρ*, may be calculated as,

13 $$ \rho =\frac{{\displaystyle {\sum}_{\mathit{\mathsf{i}}}\left({\mathit{\mathsf{x}}}_{\mathit{\mathsf{i}}}-\overline{\mathit{\mathsf{x}}}\right)\left({\mathit{\mathsf{y}}}_{\mathit{\mathsf{i}}}-\overline{\mathit{\mathsf{y}}}\right)}}{\sqrt{{\displaystyle {\sum}_i{\left({\mathit{\mathsf{x}}}_{\mathit{\mathsf{i}}}-\overline{\mathit{\mathsf{x}}}\right)}^2\;{{\displaystyle {\sum}_i\left({\mathit{\mathsf{y}}}_{\mathit{\mathsf{i}}}-\overline{\mathit{\mathsf{y}}}\right)}}^2}}}. $$

We calculated the correlation between our output variable, the cumulative number of human cysticercosis cases, and a given model parameter as linear combination between $\left({\mathit{\mathsf{x}}}_{\mathit{\mathsf{j}}}-{\widehat{\mathit{\mathsf{x}}}}_{\mathit{\mathsf{j}}}\right)$ and $\left(\mathit{\mathsf{Y}}\mathit{\hbox{-}}\widehat{\mathit{\mathsf{Y}}}\right)$, where *X*_*j*_ is the rank-transformed, sampled *jth* input parameter and *Y* our rank-transformed state variable. For *k* samples we calculate ${\widehat{X}}_{\mathit{\mathsf{j}}}$ and $\widehat{Y}$ with the following linear regression models,

14 $$ {\widehat{\mathit{\mathsf{X}}}}_{\mathit{\mathsf{j}}}={\mathit{\mathsf{c}}}_{\mathsf{0}}+{\displaystyle \sum_{\begin{array}{l}\mathit{\mathsf{p}}=1\\ {}\mathit{\mathsf{p}}\ne j\end{array}}^{\mathit{\mathsf{k}}}{\mathit{\mathsf{c}}}_{\mathit{\mathsf{p}}}{\mathit{\mathsf{X}}}_{\mathit{\mathsf{p}}}} $$

15 $$ \widehat{\mathit{\mathsf{Y}}}={\mathit{\mathsf{b}}}_{\mathsf{0}}+{\displaystyle \sum_{\begin{array}{l}\mathit{\mathsf{p}}=1\\ {}\mathit{\mathsf{p}}\ne j\end{array}}^{\mathit{\mathsf{k}}}{\mathit{\mathsf{b}}}_{\mathit{\mathsf{p}}}{\mathit{\mathsf{X}}}_{\mathit{\mathsf{p}}},} $$

with *b*_0_ and *c*_0_ the model intercepts and *b*_*p*_ and *c*_*p*_ the regression coefficients.

Together, the LHS and PRCC allow a computationally efficient sensitivity analysis to be performed for a large number of parameters [37, 38]. The significance of the influence on model output of uncertainty surrounding a parameter may be quantified by calculating the following *T* value,

16 $$ \mathit{\mathsf{T}}=\rho \sqrt{\frac{\left( N-2-\mathit{\mathsf{q}}\right)}{1-{\rho}^2}}, $$

where *N* is the sample size (the number of intervals per parameter) and *q* is the number of parameters for which we adjust (the extra, influential model parameters whose effect we wish to control for). The *T* statistic follows a Student’s *t* distribution with (*N*-2-*q*) degrees of freedom [39].

The sensitivity of the output variable of interest in relation to the intervention effectiveness (efficacy × coverage) was assessed univariately. All model parameters were held at the standard, nominal value; the intervention effectiveness/influence was sampled 1000 times from a distribution of plausible values. For pig MDA the proportion transferred form the infected pig compartments to the susceptible/immune compartments was varied from 1 (best: 100% coverage × 100 efficacy) to 0.375 (worst: 50% coverage × 75% efficacy) and the proportion of pigs treated that was assumed to have acquired natural immunity varied from 1 (best) to 0 (worst). For the pig vaccination the proportion transferred from the susceptible to recovered/immune compartments was varied from to 1 (best: 100% coverage × 100 efficacy) to 0.375 (worst: 50% coverage × 75% efficacy). For the improved animal husbandry the egg-pig transmission coefficient (*τ*) was reduced by 50% (best) or 10% (worst). For improved sanitation the egg production rate (*δ*) was reduced by 50% (best) or 10% (worst). For improved meat inspection the rate of humans contacting infected pork with a low cyst burden (*β*_*L*_) was reduced by 50% (best) or 10% (worst) and the rate of humans contacting infected pork with a high cyst burden (*β*_*H*_) was reduced by 80% (best) or 20% (worst). For the test & treat scenario the proportion of humans with taeniasis (and not with cysticercosis) transferred from the *I*_*T*_ compartment to the *S* compartment was varied from 1 (best: 100% coverage, 100% efficacy and 100% sensitivity of diagnostic test to *T. solium* and 100% specificity of the test to cysticercosis infection) to 0.3375 (worst: 50% coverage, 75% drug efficacy, 90% sensitivity of test to *T. solium* and 95% specificity of the test to cysticercosis infection).

### The basic reproduction number, *R*_0_, of taeniasis

An expression for *R*_0_ (the number of new taeniasis cases generated by a single human with taeniasis in an otherwise susceptible population) was derived using the next generation matrix approach detailed in [40–42],

17 $$ {\mathit{\mathsf{R}}}_0=\sqrt[3]{\frac{\tau \delta \left({\beta}_{\mathit{\mathsf{H}}}-{\beta}_{\mathit{\mathsf{H}}}\theta +{\beta}_{\mathit{\mathsf{L}}}\theta \right)}{{\mathit{\mathsf{d}}}_{\mathit{\mathsf{E}}}{\mathit{\mathsf{d}}}_{\mathit{\mathsf{P}}}\left(\left(1+\mathit{\mathsf{r}}\right)\theta +{\mathit{\mathsf{d}}}_{\mathit{\mathsf{H}}}+\alpha \right)}\left(1+\frac{\left(1+\mathit{\mathsf{r}}\right)\theta}{\eta +{\mathit{\mathsf{d}}}_{\mathit{\mathsf{H}}}+\alpha}\right) H}, $$

where *H* is the human population size, *r* is *r*_Cysticercosis_ as listed in Table 1 and all remaining parameters have been described above. The next generation matrix approach assesses *R*_0_ as a demographic process (the cubic root indicating that the life-cycle of the infection encompasses three components: the human host, the pig host, and the environment), analysing the acquisition of new infections over successive generations [42]. Further details of the calculation of *R*_0_ using this approach are given in Additional file 1.

Estimates of *R*_0_ were made by assessing 10,000 LHS samples from the plausible range of parameter estimates. We present the median and 2.5% and 97.5% quantiles as our best estimate and 95% credible interval (95% CrI) respectively.

## Results

### Model

**Fig. 2:**
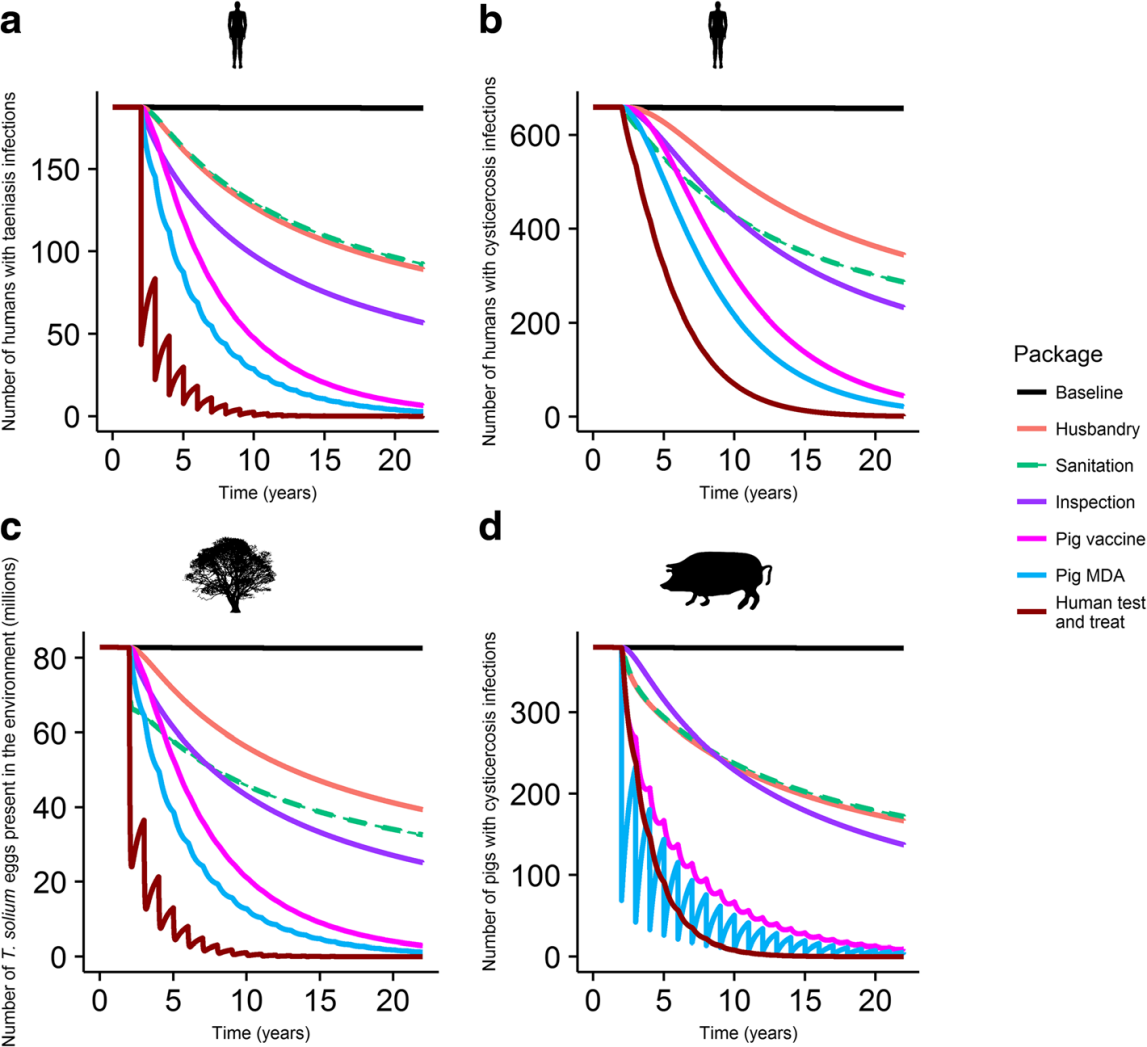
Impact of single interventions on: **a** the prevalence of taeniasis in humans, **b** the prevalence of cysticercosis in humans, **c** the number of eggs in the environment, and **d** the prevalence of cysticercosis in pigs. The *black* horizontal line represents the infection levels according to the baseline model, run at stable equilibrium in the absence of any intervention; the *orange* line represents enhanced husbandry; the *green* dashed line improved sanitation, the *purple* line improved meat inspection; the *mauve* line vaccination of the pig population, the *blue* line pig mass drug administration (MDA) and the dark *red* line human test & treat

EPICYST model outputs for the baseline (no intervention) and six implemented single intervention scenarios are shown in Fig. 2. All interventions, when applied singly, demonstrated an ability to reduce the prevalence of taeniasis and cysticercosis in humans (Fig. 2a and b, respectively), the number of eggs in the environment (Fig. 2c) and the prevalence of cysticercosis in pigs (Fig. 2d).

**Fig. 3:**
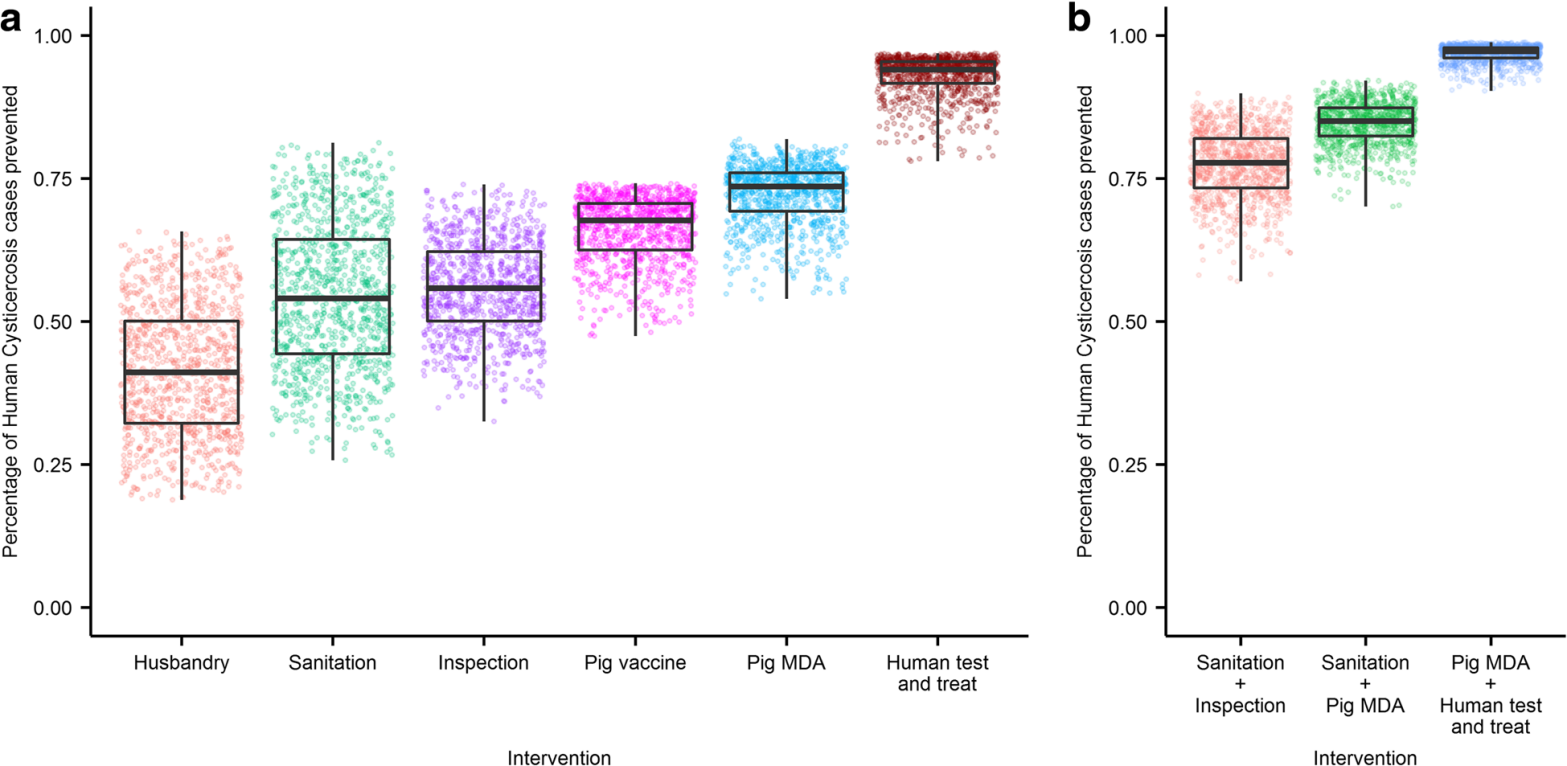
Impact of interventions on the number of human cysticercosis cases. Single **a** and the most effective **b** pairwise combinations of interventions from the behavioural, pig-targeted and human categories (all pairwise combinations are included in the SI). Intervention colours as in Fig. 2. Box and whiskers represent the range of impact estimates from 1000 sensitivity draws of intervention efficacy parameters, the midline represents the median impact, the hinges the 25^th^ and 75^th^ percentiles and whiskers the range. Points show individual run output. Due to the large amount of uncertainty in parameters estimates, the impact of parameter estimates was explored separately (see Fig. 4)

A summary of the effect of single and pair-wise combinations of interventions on the primary outcome measure of interest, the cumulative number of cysticercosis cases averted, is shown in Fig. 3. For single interventions, the human (test & treat) treatment scenario was highly effective. We estimate that annual implementation of the test & treat approach, targeting all people with taeniasis but not cysticercosis, would avert a median equal to 94% (95% credible interval, 95% CrI: 83–97%) of human cysticercosis cases.

The pig-targeted interventions had a greater impact [median % of human cysticercosis cases averted: pig MDA 74% (95% CrI: 59–80%) and pig vaccination: 68% (95% CrI: 52–73%)] than the improved husbandry, sanitation or meat-inspection interventions [median % of human cysticercosis cases averted: husbandry 41% (95% CrI: 22–62%), sanitation 54% (95% CrI: 32–77%) and meat-inspection 56% (95% CrI: 40–71%)]. Deploying the human test & treat intervention in combination with any other intervention resulted in relatively small marginal gains in the number of human cysticercosis cases averted. Combinations of behavioural and/or pig-targeted interventions were considerably more effective when paired than when applied singly. The combination of improved sanitation and meat-inspection measures had the greatest suppressive effect on infection prevalence of the strategies that were not drug- or vaccine-based [median % of human cysticercosis cases averted: sanitation + inspection 78% (95% CrI: 65–87%)]. The impact of all pairwise combination is further detailed in Additional file 1 (Fig. S1).

Pig MDA and pig vaccination produced contrasting dynamics in the infected pig compartments (Fig. 2d). Vaccination effected a much more smooth decline in the numbers of infected pigs with respect to time than MDA (Fig. 2d).

Improved sanitation and improved animal husbandry produced equivalent effects on the prevalence of taeniasis in humans and of cysticercosis in pigs (Fig. 2a, d), but contrasting effects in the prevalence of humans cysticercosis cases and the number of *T. solium* eggs in the environment (Fig. 2b, c).

### The basic reproduction number of taeniasis

For the wide range of plausible parameter values shown in Table 2, the (median) estimate for *R*_0_ was 1.4 (95% CrI: 0.5–3.6).

### Sensitivity analysis

**Fig. 4:**
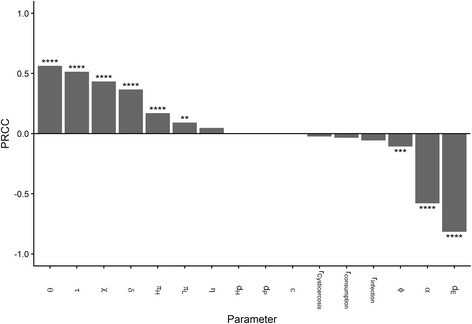
Sensitivity of model output to parameter values and their uncertainty. The metrics used is the partial rank correlation coefficient (PRCC) as described in the Main Text. ***P ≤ 0.001; ****P ≤ 0.0001

The sensitivity analysis indicated that of the 16 model parameters, 8 of them, and their associated uncertainty, were particularly influential on model output. A summary of the calculated PRCC values for all parameters in the baseline model is presented in Fig. 4. Parameters $\alpha, {\mathit{\mathsf{d}}}_{\mathit{\mathsf{E}}},\delta, \chi, \theta, \tau, \phi$ and *π*_*L*_ had highly statistically significant relationships with the outcome of interest, in part reflecting the high degree of uncertainty surrounding some parameter estimates. The parameters *r*_consumption_ and *r*_infection_, for which very little is known, had little relative influence on the outcome.

The test & treat of humans with taeniasis and the pig-focussed interventions (MDA and vaccination) proved much more robust in respect to changes in the potential coverage or efficacy of the intervention in comparison to improved husbandry, sanitation and meat-inspection interventions (Fig. 3).

## Discussion

In response to the call by the WHO for developing a validated strategy for the control and elimination of *T. solium* taeniasis/cysticercosis [1], and to the research priorities highlighted by the WHO for zoonoses and marginalized infections [6], we have developed EPICYST, a transmission model to explore in-depth the transmission dynamics of the taeniasis/cysticercosis disease system and assess a range of interventions applied singly or in combination.

Cysticercosis infections in humans are specifically modelled, capturing this clinically-relevant aspect of the parasite’s life history. The extra complexity associated with incorporating human cysticercosis into the model, as illustrated by the model diagram (Fig. 1) and example output (Fig. 2), is an important inclusion. Output from the model indicates that the prevalence of human cysticercosis is non-linearly associated with the presence of taeniasis in humans, highlighting the importance of specifically modelling the human cysticercosis dynamics. Model outputs also highlight complex interactions between specific interventions, the prevalence of taeniasis and the prevalence of cysticercosis. For example, improved sanitation and animal husbandry are projected to have very similar effects on porcine cysticercosis and human taeniasis but different dynamics with regard to human cysticercosis; sanitation impacts prevalence of human cysticercosis as it decreases the probability of human-egg contact, animal husbandry does not (Fig. 2b). This example also highlights the benefits of including an environmental compartment for eggs in the model as it facilitates the implementation of targeted interventions that impact the parasite’s life-cycle at very specific points. We have hoped to make this model flexible in other ways to a wide range of potential baseline and intervention scenarios. Inclusion of a mixture of density- and frequency-dependent rates gives the model a degree of flexibility to cope appropriately with scenarios covering a range of human and pig population densities.

The hypothetical human test & treat approach was extremely effective. However it must be stressed that this is currently still a speculative (and optimistic) assessment of a potential intervention. Although there currently are tests for detection of stage-specific human *T. solium* taeniasis antibodies and neurocysticercosis antibodies [43] that have been used in a number of field studies [44, 45], barriers exist to the introduction and financing of such an intervention on a large scale. The efficacy of a human test & treat strategy would also be influenced by the level of (taeniasis-cysticercosis) co-infection in the community, with high levels of co-infection hampering success.

The assessment of alternative approaches to human chemotherapy shows that the pig-targeted interventions, MDA and vaccination of pigs, are the most effective to reduce the prevalence of human cysticercosis in a short period. Both of these interventions, when applied annually at high coverage and assuming high efficacy reduced the median number of potential new cysticercosis cases by > 65% (Fig. 3). Pig MDA performed marginally better than pig vaccination averting additional cases over pig vaccination. The effect of the vaccine is less for two reasons: (i) there is a period prior to full vaccination of around 3 months in which pigs may become infected; and (ii) there is a small difference between the two interventions in lag times between intervention implementation and intervention effect. Pig MDA is assumed to clear immediately most of the infectious reservoir in pigs, whilst the pig infectious reservoir clears as pigs die or are slaughtered and is not replenished with the vaccination intervention. In settings where it may be difficult to achieve high coverage in a cost-effective manner, either of these interventions would be capable of having extremely positive public health effects through annual implementation. Applying pig MDA or pig vaccination in conjunction with each other or with any of the other interventions considered resulted in small marginal gains as the performance of the pig-focussed interventions when applied on their own was already very good. Any programmatic decisions to implement either human test & treat, vaccination or MDA campaigns would need to consider the long-term sustainability of such an effort, as cessation without achieving elimination would likely lead to return to pre-intervention equilibrium prevalence levels.

The potential ability of improved animal husbandry, sanitation and meat inspection to reduce the prevalence of taeniasis and cysticercosis was also demonstrated. The suppressive effect on disease prevalence was less pronounced than that of the human- and pig-targeted interventions but would still be of considerable public-health benefit. Application of these interventions in combination resulted in increased effectiveness over their use singly; the combination of improved meat inspection and sanitation was of similar effectiveness to a pig vaccination campaign but less effective than human test & treat in the scenarios modelled (Fig. 3b). The wide-ranging public health benefits associated with infrastructure-based interventions especially must not be considered in isolation. Improved sanitation infrastructure would also greatly benefit efforts to combat other severe conditions including diarrhoeal disease [46] and other helminth infections [47]. Combinations of MDA of pigs or their vaccination and improved sanitation resulted in high levels of impact against taeniasis and cysticercosis and would complement a “One Health” approach [48].

We have analysed the model to derive an equation for the basic reproduction number of taeniasis. The calculation summarises the relevant rates and processes that are responsible for the dynamics of the parasite throughout its life-cycle. The median estimate was a value of 1.4 (95% CrI: 0.5–3.6), slightly lower than the estimate derived from the previous model (1.75 [28]). The wide confidence (credible) interval around the value estimated in this paper is another reflection of the high level of uncertainty surrounding a number of parameters in the model. This expression provides us with a guide from which we can determine the threshold of extinction/persistence in the theoretical (deterministic) system. Furthermore, we can explore the relationship between individual parameters and this threshold. For instance, based on our model output and assumptions, we observe that a number of parameters (e.g. *α*, the human recovery rate from taeniasis and, *d*_*E*_, the egg removal/death rate) are non-linearly associated with *R*_0_, and may warrant further attention.

The sensitivity analysis is an extremely important component of this analysis and, it must be stressed that all conclusions drawn from model outputs are made in the presence of considerable uncertainty surrounding a number of important model parameters. This lack of data is, unfortunately, a common problem and one highlighted by the Disease Reference Group on Zoonoses and Marginalised Infectious Diseases [49]. Although a number of studies have approached the problem of estimating the persistence of eggs in the environment [50–54], the removal rate of eggs, *d*_*E*_, remains a highly uncertain and influential parameter. Furthermore, this parameter is likely to be most strongly influenced by external meteorological, ecological and seasonal factors not included in this model. As expected, the transmission parameters, *θ* and *τ*, and *ϕ*, are all influential on model output and also likely to be heterogeneous across a wide range of epidemiological scenarios. The influence of the human recovery rate from taeniasis, *α*, is also statistically significant. Pig-focussed interventions are also sensitive to model assumptions regarding the acquisition of immunity.

From the above, we identify key gaps in the fundamental scientific knowledge that need to be addressed for more accurate parameterisation of transmission models of taeniasis/ cysticercosis, namely: (i) the persistence of *T. solium* eggs in the environment with respect to climate and seasonality; (ii) the average lifespan of the adult worm in the human host (the reciprocal of *α*); and (iii) the acquisition of immunity to infection in the pig population.

The sensitivity of projected intervention impact has also been assessed. Pig MDA, pig vaccination and human test & treat appear to be robust to variability in assumed intervention coverage and efficacy relative to the other singly implemented interventions. There are larger uncertainties surrounding the efficacy and effect size of the non-chemotherapeutic interventions than for anthelmintic treatment or vaccination, and this is highlighted in this analysis.

### Limitations

In addition to uncertainty surrounding parameter estimates, we have identified a number of other limitations. The model does not specifically include the development of neurocysticercosis in human patients with cysticercosis. This stage was not included due to a lack of data but is of critical importance due to the clinical implications of neurocysticercosis. Behavioural change is particularly hard to parameterise; this is reflected in the wide range of parameters that are associated with human behaviours in the sensitivity analysis. For the same reason, we did not model an educational intervention. Conducting studies that quantify the impact of such interventions on transmission remains an important research gap. We have made simplifying assumptions regarding a number of potential heterogeneities in the system; there is no age structure or heterogeneity of risk in humans and any spatial or seasonal elements have been omitted. We assessed interventions under conditions pertaining to a specific endemic state and generalisations of results are presented with this in mind. We have developed a deterministic model which may not be suitable for assessing targets of disease elimination, when stochastic elements become increasingly important. In future work, stochastic approaches such as those demonstrated in previous models [28], will be explored, as we are planning to develop an individual-based analogue of EPICYST. However, the validity of models with increased complexity will depend critically on an increase in available data for parameterisation. It will be important to fit and validate the model with field data and longitudinal intervention studies, preferably from a wide range of epidemiological scenarios.

## Conclusions

We are able to make a number of recommendations to facilitate progress towards the 2015 and the 2020 goals laid out in the WHO NTD roadmap [2] and that support those made by an expert panel to the WHO in 2011 [55]. Firstly, the potential for a human test & treat strategy to be highly effective supports calls for the prioritisation of further research into the development of affordable, fast and accurate point-of-care diagnostics to distinguish between taeniasis and cysticercosis [43–45]. Secondly, pig MDA or pig vaccination is capable of effecting a significant impact on averting human cysticercosis at present, warranting their further evaluation in large-scale field trials. Thirdly, where a more integrated approach to public and veterinary health is called for, combinations of improved animal husbandry, sanitation and meat inspection may be preferable.

In addition, we have identified priority areas for further research for the improvement of taeniasis/cysticercosis transmission models, including the persistence of *T. solium* eggs in the environment, the duration of adult worm lifespan, and the acquisition of pig immunity to infection.

The model allows scenario-specific implementation of a range of interventions or combinations of interventions, of which a non-exhaustive list have been explored. We hope that the model presented will be a valuable tool for scientists and policy makers to guide future research efforts and inform policy, in addition to acting as a foundation for future modelling developments and providing a framework for the assessment of the cost-effectiveness of interventions [23].

## Additional file

Additional file 1: Derivation of an expression for the basic reproduction number, *R*_0_, for taeniasis in humans. **Figure S1:** Impact of pairwise combinations of interventions on the number of human cysticercosis cases. Box and whiskers represent the range of impact estimates from 1000 sensitivity draws of efficacy parameters, the midline represents the median impact, the hinges the 25^th^ and 75^th^ percentiles and whiskers the range. Points show individual run output. Due to the large amount of uncertainty in parameters estimates, the impact of parameter estimates was explored separately. (DOCX 2105 kb)

## Abbreviations

95% CI: 95% confidence interval
95% CrI: 95% credible interval
LHS: Latin-hypercube sampling
MDA: Mass drug administration
NTD: Neglected tropical disease
PCT: Preventative chemotherapy
PRCC: Partial rank correlation coefficient
*R*_0_: Basic reproduction number or ratio
SAE: Severe adverse effect
WHO: World Health Organization.
