# Supplementary material for: Assessing the impact of intervention strategies against Taenia solium cysticercosis using the EPICYST transmission model
Source: Parasit Vectors. 2017 Feb 9;10:73. doi: 10.1186/s13071-017-1988-9 (PMC5301381; doi:10.1186/s13071-017-1988-9)
Supplement: Additional file 1: — Derivation of an expression for the basic reproduction number, R 0, for taeniasis in humans. Figure S1: Impact of pairwise combinations of interventions on the number of human cysticercosis cases. Box and whiskers represent the range of impact estimates from 1000 sensitivity draws of efficacy parameters, the midline represents the median impact, the hinges the 25th and 75th percentiles and whiskers the range. Points show individual run output. Due to the large amount of uncertainty in parameters estimates, the impact of parameter estimates was explored separately. (DOCX 2105 kb) [file 13071_2017_1988_MOESM1_ESM.docx]

# Additional File 1

Derivation of an expression for the basic reproduction number, *R*_0_, for taeniasis in humans

Under the prevalence framework we define *R*_0_ as the average number of cases (humans with taeniasis) that a single case would generate in a fully susceptible population. A next generation matrix approach following [1–3] was used to derive an expression for *R*_0_. Equations are defined in terms of proportions; otherwise, the notation follows the one presented in the Main Text and its Table 1. Equations relating only to infected compartments are used,

$\frac{dI_{T}}{dt}=\beta_{L}SI_{PL}+\beta_{H}SI_{PH}-\alpha I_{T}-(1+r)\theta I_{T}E-d_{H}I_{T}$ (S1)

$\frac{dI_{TC}}{dt}=\beta_{L}I_{C}I_{PL}+\beta_{H}I_{C}I_{PH}+(1+r)\theta I_{T}E-\alpha I_{TC}-\eta I_{TC}-d_{H}I_{TC}$ (S2)

$\frac{dE}{dt}=\delta I_{T}+\delta I_{TC}-d_{E}E$ (S3)

 (S4)

 (S5)

Define the *F* matrix

Define the *V* matrix (assuming *E*=1)

$$\begin{matrix} V_{1}=\alpha I_{T}+d_{H}I_{T}+(1+r)\theta I_{T}E \\ V_{2}=\alpha I_{TC}+\eta I_{TC}+d_{H}I_{TC}-(1+r)\theta I_{T}E \\ V_{3}=d_{E}E \\ V_{4}=d_{P}I_{PL} \\ V_{5}=d_{P}I_{PH} \end{matrix}$$

$$\underline{V}=\left[\frac{\partial V_{i}(x_{0})}{\partial(x_{j})}\right]=\left[\begin{matrix} \alpha+d_{H}+(1+r)\theta& 0 & 0 & 0 & 0 \\ -(1+r)\theta& \alpha+\eta+d_{H} & 0 & 0 & 0 \\ 0 & 0 & d_{E} & 0 & 0 \\ 0 & 0 & 0 & d_{P} & 0 \\ 0 & 0 & 0 & 0 & d_{P} \end{matrix}\right]$$

Invert the *V* matrix

$$\underline{V}^{-1}=\left[\begin{matrix} \frac{1}{\alpha+d_{H}+(1+r)\theta} & 0 & 0 & 0 & 0 \\ \frac{(1+r)\theta}{(\alpha+d_{H}+(1+r)\theta)(\alpha+\eta+d_{H})} & \frac{1}{\alpha+\eta+d_{H}} & 0 & 0 & 0 \\ 0 & 0 & \frac{1}{d_{E}} & 0 & 0 \\ 0 & 0 & 0 & \frac{1}{d_{P}} & 0 \\ 0 & 0 & 0 & 0 & \frac{1}{d_{P}} \end{matrix}\right]$$

Multiple the $\underline{F}$ matrix by the inverted $\underline{V}$ matrix, $\underline{V}^{-1}$


*R*_0_ is the dominant eigenvalue of this matrix (det is the determinant and *I* the identity matrix),

$\det(\underline{F}\cdot\underline{V}^{-1}-\lambda I)=0$ (S6)

where$\underline{F}\cdot\underline{V}^{-1}-\lambda I$ is,

Therefore,

 (S7)

Setting $I_{c}=0$ and $S_{p}=S=1$ gives

$-\lambda^{5}+\lambda^{2}\left[\frac{\delta\tau(\beta_{H}-\beta_{H}\theta+\beta_{L}\theta)}{d_{E}d_{P}((1+r)\theta+d_{H}+\alpha)}\left(1+\frac{(1+r)\theta}{\eta+d_{H}+\alpha}\right)\right]=0$ (S8)


 (S9)

## References

1. Diekmann O, Heesterbeek JAP, Roberts MG. The construction of next-generation matrices for compartmental epidemic models. J R Soc Interface. 2010;7(47):873–885.

2. Heffernan JM, Smith RJ, Wahl LM. Perspectives on the basic reproductive ratio. J R Soc Interface. 2005;2(4):281–293.

3. Van den Driessche P, Watmough J: Reproduction numbers and sub-threshold endemic equilibria for compartmental models of disease transmission. Math Biosci. 2002;180:29–48.

**Additional Figure S1. Impact of pairwise combinations of interventions on the number of human cysticercosis cases**

Box and whiskers represent the range of impact estimates from 1000 sensitivity draws of efficacy parameters, the midline represents the median impact, the hinges the 25^th^ and 75^th^ percentiles and the whiskers the range. Points show individual run output. Due to the large amount of uncertainty in parameters estimates, the impact of parameter estimates was explored separately.
